# Masitinib Combined with Standard Gemcitabine Chemotherapy: *In Vitro* and *In Vivo* Studies in Human Pancreatic Tumour Cell Lines and Ectopic Mouse Model

**DOI:** 10.1371/journal.pone.0009430

**Published:** 2010-03-04

**Authors:** Martine Humbert, Nathalie Castéran, Sébastien Letard, Katia Hanssens, Juan Iovanna, Pascal Finetti, François Bertucci, Thomas Bader, Colin D. Mansfield, Alain Moussy, Olivier Hermine, Patrice Dubreuil

**Affiliations:** 1 AB Science SA, Paris, France; 2 Inserm U891, Centre de Recherche en Cancérologie de Marseille, Signalisation, Hématopoïèse et Mécanismes de l'Oncogenèse, Centre de Référence des Mastocytoses, Marseille, France; 3 Institut Paoli-Calmettes, Marseille, France; 4 Université de la Méditerranée, Marseille, France; 5 Inserm U624, Stress Cellulaire, Parc Scientifique et Technologique de Luminy, Marseille, France; 6 Inserm, U891, Centre de Recherche en Cancérologie de Marseille, Département d'Oncologie Moléculaire, Département d'Oncologie Médicale, Institut Paoli-Calmettes, Marseille, France; 7 Hôpital Necker, Service d'Hématologie, CNRS UMR 8147, Centre de Référence des Mastocytoses, Université Paris V René Descartes, Paris, France; Dr. Margarete Fischer-Bosch Institute of Clinical Pharmacology, Germany

## Abstract

**Background:**

Tyrosine kinases are attractive targets for pancreatic cancer therapy because several are over-expressed, including PDGFRα/β, FAK, Src and Lyn. A critical role of mast cells in the development of pancreatic cancer has also been reported. Masitinib is a tyrosine kinase inhibitor that selectively targets c-Kit, PDGFRα/β, Lyn, and to a lesser extent the FAK pathway, without inhibiting kinases of known toxicities. Masitinib is particularly efficient in controlling the proliferation, differentiation and degranulation of mast cells. This study evaluates the therapeutic potential of masitinib in pancreatic cancer, as a single agent and in combination with gemcitabine.

**Methodology/Findings:**

Proof-of-concept studies were performed *in vitro* on human pancreatic tumour cell lines and then *in vivo* using a mouse model of human pancreatic cancer. Molecular mechanisms were investigated via gene expression profiling. Masitinib as a single agent had no significant antiproliferative activity while the masitinib/gemcitabine combination showed synergy *in vitro* on proliferation of gemcitabine-refractory cell lines Mia Paca2 and Panc1, and to a lesser extent *in vivo* on Mia Paca2 cell tumour growth. Specifically, masitinib at 10 µM strongly sensitised Mia Paca2 cells to gemcitabine (>400-fold reduction in IC_50_); and moderately sensitised Panc1 cells (10-fold reduction). Transcriptional analysis identified the Wnt/β-catenin signalling pathway as down-regulated in the cell lines resensitised by the masitinib/gemcitabine combination.

**Conclusions:**

These data establish proof-of-concept that masitinib can sensitise gemcitabine-refractory pancreatic cancer cell lines and warrant further *in vivo* investigation. Indeed, such an effect has been recently observed in a phase 2 clinical study of patients with pancreatic cancer who received a masitinib/gemcitabine combination.

## Introduction

Pancreatic cancer is an aggressive malignancy that is frequently diagnosed at an advanced stage with poor prognosis. In approximately 15–20% of cases the tumour is resectable, but only 20% of these patients will survive 5 years [1]. For locally advanced, unresectable and metastatic disease, chemo- and radiotherapy provide relatively little benefit. Gemcitabine (Gemzar, Lilly France), a nucleoside analogue of cytidine, improves symptoms and survival as compared to 5­FU-based chemotherapy, and is now established as the standard systemic treatment in pancreatic cancer [2]. However, the efficacy of gemcitabine as a single agent remains modest, with a median survival of approximately 6 months in randomised trials and a 12-month survival of ≤20% [3], [4]. A number of clinical trials are currently underway to explore the combination of gemcitabine with either cytotoxic and/or biological targeted compounds. So far, results have been disappointing, showing no or little benefit compared to gemcitabine monotherapy [5], [6]. In addition, there are numerous side-effects associated with gemcitabine including myelosuppression. Consequently, the development of less toxic and more efficient treatment strategies is critical to improve the clinical management and prognosis of these patients.

The causes of pancreatic cancer are not well understood but attention is increasingly being directed towards the role of growth factors. Several growth factors and their receptors are over-expressed during the progression of pancreatic cancer, such as epithelial growth factor (EGF), platelet-derived growth factor (PDGF), fibroblast growth factor (FGF), and vascular endothelial growth factor (VEGF) [7]. Deregulated expression of cytoplasmic tyrosine kinases has also been associated with poor prognosis and chemoresistance. In particular, gemcitabine resistance in pancreatic cancer is often associated with high expression of focal adhesion kinase (FAK) [8], a protein involved in metastasis; and elevated expression and activity of Src Family Kinases (SFK), including SRC and Lyn, have also been reported in numerous human cancer cell lines and tumour tissues [9]–[11]. Moreover, increasing evidence indicates that recruitment of inflammatory cells, especially infiltration by mast cells, facilitates the growth and spread of cancer via the production of molecules that enhance tumour invasiveness. This connection has been made for both exocrine (ductal adenocarcinomas [12]) and endocrine pancreatic cancers (islet tumour cancers [13]). Therefore, inhibition of mast cell function may prove to be therapeutically useful in restraining the growth of pancreatic cancer.

Masitinib is a novel tyrosine kinase inhibitor (TKI) that specifically and selectively targets various isoforms of the c-Kit receptor, including wild-type and those with constitutively active c-Kit mutations in the extracellular or juxtamembrane domains, PDGFRα, PDGFRβ, Lyn, and to a lesser extent FGFR3 and the FAK pathway [14]. Due to its activity against c-Kit and Lyn, masitinib is particularly efficient at controlling the proliferation, differentiation and degranulation of mast cells. Masitinib's antimastocyte potential is demonstrated through its efficacy in canine mast cell tumours [15], and rheumatoid arthritis in humans [16]. Hence, given the reported expression of PDGFRβ and c-Kit in pancreatic cancer [17], the implication of mast cells in pancreatic cancer development, and association of FAK with chemoresistance, it is hypothesised that masitinib may be of therapeutic potential in this disease. This study evaluated masitinib using *in vitro* and *in vivo* models of human pancreatic cancer, both as a single agent and in combination with gemcitabine, with the objective of establishing proof-of-concept. Molecular mechanisms were investigated via gene expression profiling.

## Materials and Methods

### Reagents and Cancer Cell Lines

Masitinib (AB Science) was prepared from powder as a 10 or 20 mM stock solution in dimethyl sulfoxide and stored at −80°C. Gemcitabine was obtained as a powder and dissolved in sterile 0.9% NaCl solution and stored as aliquots at −80°C. Fresh dilutions were prepared for each experiment.

Pancreatic cancer cell lines (Mia Paca­2, Panc­1, BxPC-3 and Capan-2) were obtained from Dr. Juan Iovanna (Inserm, France) [18]. Cells were maintained in RPMI (BxPC-3, Capan-2) or DMEM (Mia Paca­2, Panc­1) medium containing Glutamax-1 (Lonza), supplemented with 100 U/ml penicillin, 100 µg/ml streptomycin, and 10% foetal calf serum (FCS) (AbCys). Expression of tyrosine kinases was determined by RT-PCR using Hot Star Taq (Qiagen GmbH) in a 2720 Thermal Cycler (Applied Biosystems). All RT-PCR primer sequences used in this study are listed in the Supporting Information (Table S1).

### *In Vitro* Tyrosine Phosphorylation Assays

Mia Paca-2 cells (5×10^6^) were treated for 6 hours with increasing concentrations of masitinib in DMEM medium with 0.5% serum. Cells were then placed on ice, washed in PBS, and lysed in 200 µl of ice-cold HNTG buffer (50 mM HEPES pH 7, 50 mM NaF, 1 mM EGTA, 150 mM NaCl, 1% Triton X-100, 10% glycerol, and 1.5 mM MgCl_2_) in the presence of protease inhibitors (Roche Applied Science) and 100 µM Na_3_VO_4_. Proteins (20 µg) were resolved by SDS-PAGE 10%, followed by western blotting and immunostaining. The following primary antibodies were used: rabbit anti-phospho-GRB2 antibody (sc-255 1∶1000), and anti-phosphotyrosine antibody (4G10 1∶1000, Cell Signalling Technology, Ozyme). Primary antibodies were detected with 1∶10,000 horseradish peroxidase-conjugated anti-rabbit antibody (Jackson Laboratory) or 1∶20,000 horseradish peroxidase-conjugated anti-mouse antibody (Dako-France SAS). Immunoreactive bands were detected using enhanced chemiluminescent reagents (Pierce).

### Proliferation Assays

Cytotoxicity of masitinib and gemcitabine was assessed using a WST-1 proliferation/survival assay (Roche Diagnostics) in growth medium containing 1% FCS. Treatment was started with the addition of the relevant drug. For combination treatment (masitinib plus gemcitabine), cells were first resuspended in medium (1% FCS) containing 0, 5 or 10 µM masitinib and incubated overnight before gemcitabine addition. After 72 hours, WST-1 reagent was added and incubated with the cells for 4 hours before absorbance measurement at 450 nm in an EL800 Universal Microplate Reader (Bio-Tek Instruments Inc.). Media alone was used as a blank and proliferation in the absence of drug served as a positive control. Results are representative of three or four experiments. The masitinib sensitisation index is the ratio of the IC_50_ of gemcitabine against the IC_50_ of the drug combination.

### *In Vivo* Experiments

Male Nog-SCID mice (7 weeks old) were obtained from an internal breeding program and were housed at the animal care unit SCEA of the Centre de Recherche en Cancérologie de Marseille U891 (Marseille, France) under specific pathogen-free conditions at 20±1°C in a 12-hour light/12-hour dark cycle and *ad libitum* access to food and filtered water. This study was approved by the ethical review board at the Centre de Recherche en Cancerolgie de Marseille and carried out in compliance with the INSERM ethical guidelines of animal experimentation. The animal care unit U891 (Marseille, France) is authorised by the French Ministries of Agriculture and Research (Agreement N° B13-OSS-4). Mia Paca-2 cells were cultured as described above. At day 0 (D0), mice were injected with 10^7^ Mia Paca-2 cells in 200 µl PBS into the right flank. Tumours were allowed to grow for 1.5 to 4 weeks until the desired tumour size was reached (∼200 mm^3^). At day 28, animals were allocated into four treatment groups (n = 7 to 8 per group), ensuring that each group's mean body weight and tumour volume were well matched. Treatment was then administered for up to 4 weeks, after which time the animals were sacrificed. Treatments consisted of either: a) daily sterile water for the control group, b) an intraperitoneal (i.p.) injection of 50 mg/kg gemcitabine twice a week, c) daily gavage with 100 mg/kg masitinib, or d) combined i.p injection of 50 mg/kg gemcitabine twice a week and daily gavage with 100 mg/kg masitinib. Tumour size was measured with callipers and tumour volume was estimated using the formula: volume = (length × width^2^)/2. The tumour growth inhibition ratio was calculated as (100) × (median tumour volume of treated group)/(median tumour volume of control group).

### Statistical Analysis

Relative changes in tumour volumes were compared between treatment groups using a variance analysis (ANOVA). Normality of relative changes in tumour volumes between day 28 and day 56 was first tested using the Shapiro-Wilk test of normality. In the event of a positive treatment effect, treatment groups were compared two-by-two using Tukey's multiple comparison test. A *p*-value <0.05 was considered as significant.

### Microarray Data and Pathway Analysis

Gene expression profiling of cell lines (from 2 µg RNA) was assessed using whole-genome Affymetrix U133 Plus 2.0 human oligonucleotide microarrays. Generation of expression matrices, data annotation, filtering and processing have been previously described [19]. Microarray statistics and cluster analysis were performed by the Robust Multichip Average method in R using Bioconductor [20] and using the Cluster and TreeView programs [21]. Drug response signatures were generated by differential analysis, which compared the expression profile of each treated cell line with that of the untreated cell line by measuring the fold-change (treated/untreated) of each probe set. The lists of differential genes were interrogated using the Ingenuity Pathway Analysis software (Version 5.5.1-1002; Ingenuity Systems) with a significance threshold for the corrected *p*-value <0.05. MIAME compliant array data can be accessed at (www.ebi.ac.uk/arrayexpress) using the accession number GSE17987.

## Results

### Effect of Masitinib on Pancreatic Cancer Cells *In Vitro*

**Figure 1:**
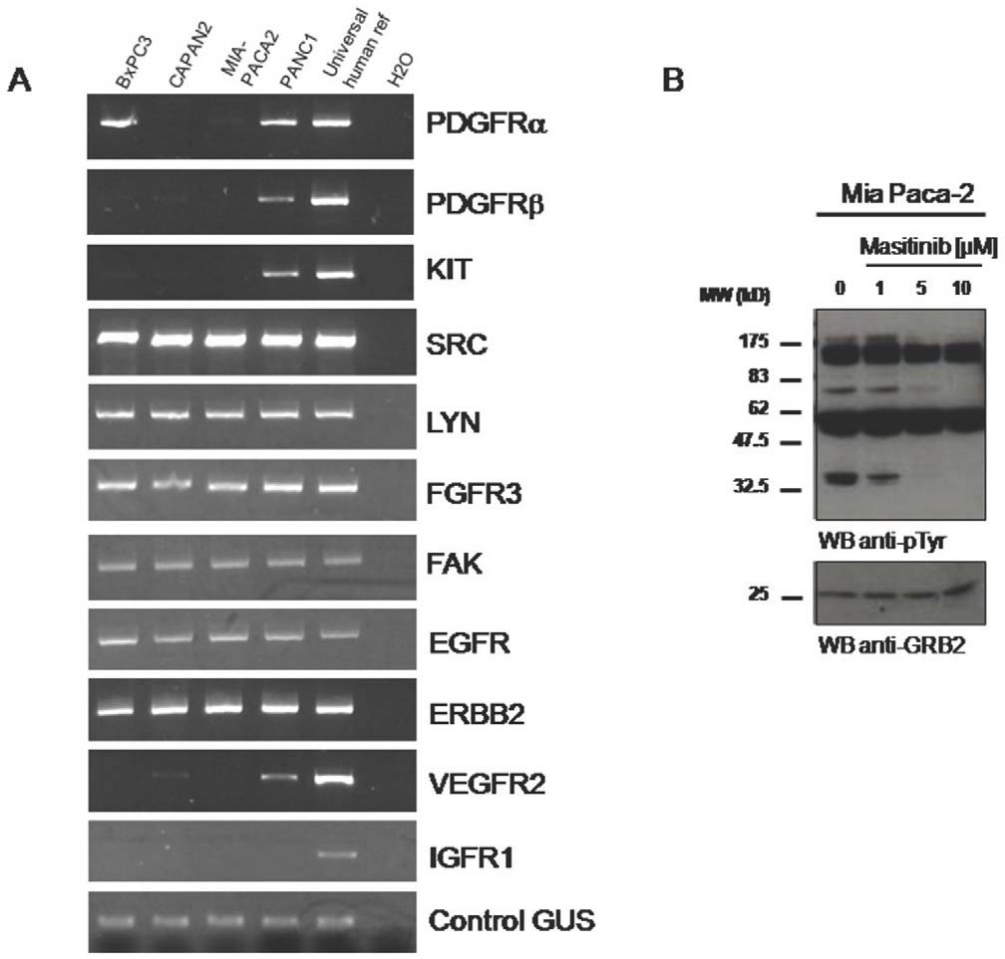
Tyrosine kinase mRNA expression profile in human pancreatic cancer cell lines. (A) Messenger RNA expression of various receptor and cytoplasmic tyrosine kinases was analysed by RT-PCR. Universal human reference total RNA was used as positive control for primers and the ubiquitous β-glucoronidase (GUS) served as an internal control for all RT-PCR reactions. (B) Tyrosine phosphorylation of proteins in response to masitinib. Mia Paca-2 cells (5×10^6^) were treated for 6 hours at 37°C with various concentrations of masitinib. Total cell lysates were prepared and tyrosine phosphorylation was analysed by western blot with antibodies against phosphotyrosine (anti-pTyr). Anti-GRB2 WB demonstrates comparable loading of proteins. MW = molecular weight.

PCR with gene-specific primers was performed to determine the expression profile of masitinib's targets in four human pancreatic cancer cell lines: Mia Paca­2, Panc­1, BxPC-3 and Capan-2. C-Kit was detectable in Panc-1 cells (described previously by Yasuda et al. [22]) but was undetectable in all the other cell lines. PDGFRα was expressed in BxPC-3 and Panc-1 cells while PDGFRβ was mainly expressed in Panc-1 cells. A broader profile of tyrosine kinases revealed strong expression of the EGFR family members ErbB1 and ErbB2, src family kinases Src and Lyn, FAK and FGFR3, in all four cell lines (Figure 1A).

To estimate the range of masitinib concentrations necessary to sensitise pancreatic tumour cell lines to chemotherapy, we assessed the ability of masitinib to block protein tyrosine phosphorylation by western blot analysis in cell lysates. Figure 1B shows a strong pattern of protein tyrosine phosphorylation at baseline in Mia Paca­2 cells. Treatment with masitinib clearly inhibited tyrosine phosphorylation at 1 µM and beyond, demonstrating that masitinib is active at these concentrations. The control protein GRB2 remained unchanged under all treatment conditions. Similar results were obtained with the three other pancreatic tumour cell lines (data not shown). Based on these results, a masitinib concentration of up to 10 µM was considered appropriate to study its effect on cell proliferation.

**Figure 2:**
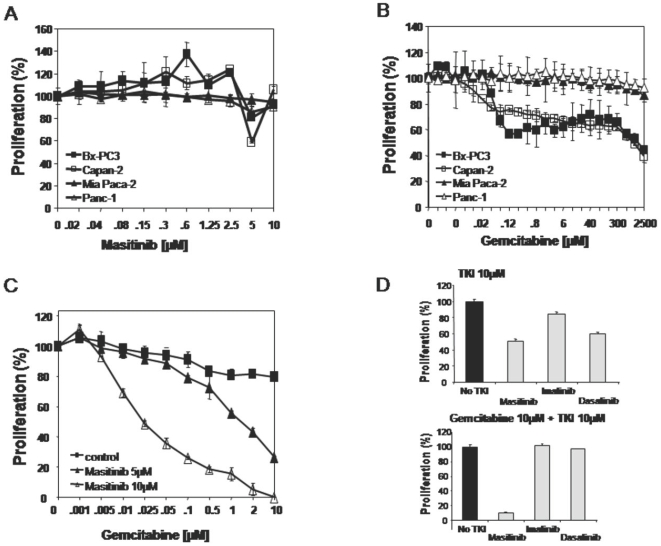
Masitinib resensitisation of resistant pancreatic tumour cell lines Mia Paca-2 and Panc-1 to gemcitabine. Sensitivity of pancreatic tumour cell lines to masitinib or gemcitabine as single agents, or in combination, was assessed using WST-1 proliferation assays. Four cell lines were tested for their sensitivity to masitinib (A) or gemcitabine (B). (C) Combination treatment of masitinib plus gemcitabine tested on gemcitabine-resistant Mia Paca-2 cells. (D) Sensitivity of resistant Mia Paca-2 cells to various tyrosine kinase inhibitors alone (top) or in combination with gemcitabine (bottom) was analysed in WST-1 proliferation assays. TKI = tyrosine kinase inhibitor.

The antiproliferative activity of masitinib or gemcitabine in monotherapy was assessed by WST-1 assays (Figures 2A and B). Masitinib did not significantly affect the growth of the tested cell lines, with an IC_50_ of 5 to 10 µM. Figure 2B shows that gemcitabine inhibits cell lines BxPC-3 and Capan-2 with an IC_50_ of 2–20 µM, while Mia Paca-2 and Panc-1 cells show resistance (IC_50_>2.5 mM) as previously reported [18]. Masitinib's potential to enhance gemcitabine cytotoxicity was assessed by pre-treating cell lines with masitinib overnight then exposing them to different doses of gemcitabine and recording the IC_50_ concentrations. Table 1 summarises the IC_50_ of gemcitabine in the absence or presence of 5 and 10 µM masitinib. Mia Paca-2 cells, pre-treated with 5 and 10 µM masitinib, were significantly sensitised to gemcitabine, as evidenced by the substantial reductions (>400-fold reduction) in gemcitabine IC_50_ (Figure 2C; Table 1). Panc-1 cells were moderately sensitised (10-fold reduction) and no synergy was observed in the gemcitabine-sensitive cell lines Capan-2 and BxPC-3 (Table 1). The treatment's antiproliferative action was confirmed via microscopic observation, which clearly revealed cells to be dying rather than being arrested in the cell cycle (data not shown). These results suggest that pre-treatment with masitinib can restore cellular responsiveness to gemcitabine.

**Table 1:** IC_50_ concentrations (µM) of various masitinib and/or gemcitabine treatment regimens in different pancreatic cell lines.

	Masitinib	Gemcitabine	Gemcitabine plus 5 µM masitinib	Gemcitabine plus 10 µM masitinib	Sensitisation Index*
BxPC-3	5–10	10	10	10	1
Capan-2	5–10	2	2	NA	1
Mia Paca-2	5–10	>10	1.5	0.025	400
Panc-1	5–10	>10	8	1	10

*Sensitisation Index is defined as the IC_50_ ratio of gemcitabine alone against the gemcitabine plus masitinib combination. NA = Not available.

### Comparison of Masitinib to Other TKIs for Their Potential to Sensitise Gemcitabine-Resistant Pancreatic Cancer Cells

Similar TKI plus gemcitabine combination experiments to those described above were performed with gemcitabine-resistant Mia Paca-2 cells to compare masitinib with imatinib (Gleevec, STI-571; Novartis), a TKI targeting ABL, PDGFR, and c-Kit); and dasatinib (Sprycel, Bristol-Myers Squibb), a TKI targeting SRC, ABL, PDGFR, and c-Kit. Mia Paca-2 cell proliferation was not inhibited by imatinib alone (10 µM), whereas it was partially inhibited in the presence of low concentrations of the SRC inhibitor dasatinib (>0.1 µM); albeit with <50% of the cells remaining resistant (Figure 2D). Pre-incubation of cells with 10 µM of imatinib or dasatinib did not result in an increased response of Mia Paca-2 cells to gemcitabine as compared to masitinib (Figure 2D). Therefore, only masitinib was able to restore sensitivity to gemcitabine in Mia Paca­2 cells.

### Effect of Masitinib on Human Pancreatic Cancer *In Vivo* in a Nog-SCID Mouse Model

**Figure 3:**
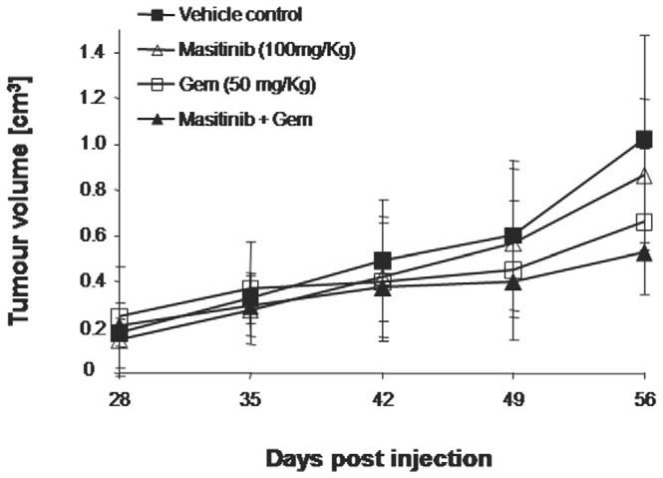
*In vivo* anti-tumour activity of masitinib in a Nog­SCID mouse model of human pancreatic cancer. Mia Paca-2 tumour cells (10^7^) were injected into the flank of Nog­SCID mice. Treatment was initiated 28 days after tumour cell injection. The different groups were treated with either: twice weekly injections of gemcitabine (i.p. 50 mg/kg), daily oral masitinib (100 mg/kg), water (control), or combined daily oral masitinib (100 mg/kg) and twice weekly injections of gemcitabine. Mice were treated for 28 days.

Preliminary experiments showed the optimal doses to use in this model (in terms of the combination's response and risk) were masitinib at 100 mg/kg/day by gavage and gemcitabine at 50 mg/kg twice weekly by i.p. injection (data not shown). Tumours of the desired size (200 mm^3^) were obtained 28 days following Mia Paca-2 cell injection. The tumour size was monitored every 7 days until day 56, after which time the animals were sacrificed. Figure 3 shows stabilisation of tumour growth between day 35 and 49 in mice treated with gemcitabine or gemcitabine plus masitinib. Tumour response for each treatment group is reported in Table 2. The antitumour effect continued until day 56 (28 days of treatment) with better control of tumour growth evident in mice treated with the gemcitabine plus masitinib combination, as compared to the masitinib monotherapy or the control groups. Overall response analysis at day 56 defined a responder as having a smaller tumour volume than the lower range limit of the control group (i.e. 711 mm^3^). Following 28 days of treatment, 3/7 mice (43%) treated with masitinib alone were responders, with 6/8 mice (75%) responding in both the gemcitabine monotherapy and masitinib plus gemcitabine groups. Median tumour volumes were significantly reduced in the gemcitabine monotherapy and masitinib plus gemcitabine groups relative to control (*p*<0.05 Tukey's multiple comparison test). Although statistical significance was not demonstrated (*p*>0.05), the combination of masitinib plus gemcitabine appeared more potent than gemcitabine alone, with this observed trend being consistent over two separate experiments.

**Table 2:** Effect of masitinib plus gemcitabine on Mia Paca-2 pancreatic tumours in Nog­SCID mice, following 28 days of treatment.

Treatment group	Response rate	Tumour volume (mm^3^)		Relative change in volume (%)	
		Median	Range	Mean±SD	Range
Control	0/7 (0%)	1023	711–1422	5.4±2.3	2.8–9.0
Masitinib (100 mg/kg)	3/7 (43%)	865	450–1543	4.8±1.4	2.6–6.6
Gemcitabine (50 mg/kg)	6/8 (75%)	662*	353–1317	2.1±1.1	0.7–3.6
Masitinib+Gemcitabine	6/8 (75%)	526*	166–1190	2.4±1.8	0.0–5.3

*p-value<0.05 versus control using Tukey's multiple comparison test. Responders are defined as having a smaller tumour volume than the lower range limit of the control group (i.e. 711 mm^3^). Relative change in tumour volume was measured from day 28 to day 56.

### Gene Expression Signature in Response to Masitinib Plus Gemcitabine Treatment

**Figure 4:**
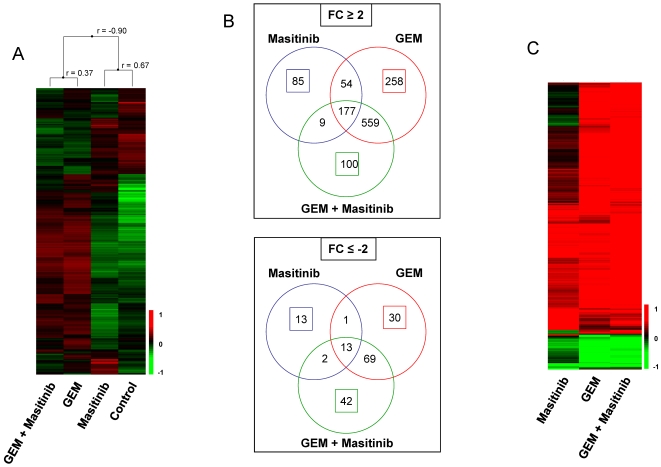
Gene expression analysis in response to masitinib, gemcitabine or combined treatment. Gene expression analysis was performed following 24 hours of treatment with masitinib 5 µM, gemcitabine 1 µM, or masitinib plus gemcitabine. (A) Hierarchical clustering of treated and untreated Mia Paca-2 cell line and 13,612 probe sets with variation expression levels across all samples. Each row represents a probe set and each column a sample. The expression level of each probe set, relative to its median abundance across the samples, is depicted according to the colour scale shown at the bottom. Red and green indicate expression levels above and below the median, respectively. The magnitude of deviation is represented by the colour saturation. The dendrogram of samples (on top of matrix) represents overall similarities in expression profiles. The correlation coefficient (r) of linked samples is indicated. (B) Venn diagram of genes differentially expressed after each treatment. Up: genes up-regulated (Fold Change: FC≥2); down: genes down-regulated (FC≤−2). The coloured circles indicate deregulated genes (specific or not to the treatment), whereas the coloured squares indicate the genes deregulated in a treatment-specific manner. (C) Hierarchical clustering of treated cell lines and 1412 genes deregulated in response to treatment in at least one condition. Legend is similar to (A). Before clustering, the expression profile of each treated cell line was divided by that of the control cells. Data are not median-centred.

To better understand the molecular mechanisms underlying the observed masitinib chemosensitisation, Mia PaCa-2 cells under various treatment regimens (untreated, masitinib monotherapy, gemcitabine monotherapy, or masitinib plus gemcitabine in combination), were profiled using DNA microarrays. Whole-genome clustering of the four cell samples sorted them into two opposite clusters (Figure 4A). The two treatment regimens with gemcitabine clustered together (left cluster), whereas cells treated with masitinib alone clustered with the untreated cells (right cluster). This result suggests that changes of gene expression in response to masitinib treatment are less numerous than those associated with gemcitabine chemotherapy, which is to be expected as masitinib is a more targeted agent. This was confirmed by the differential analysis of the expression profile (Figure 4B). Using a fold-change threshold of 2 (up-regulation) and 2 (down-regulation), we identified 971 deregulated genes after combined masitinib plus gemcitabine treatment (845 up- and 126 down-regulated); 1161 deregulated genes after gemcitabine monotherapy (1048 up- and 113 down-regulated); and only 354 deregulated genes after masitinib monotherapy (325 up- and 29 down-regulated). Results are displayed in Figure 4C as a colour-coded matrix including all 1412 deregulated genes. These drug response expression signatures were characterised via pathway analysis using Ingenuity software (see Supporting Information; Table S2). From the 971 genes deregulated after combined masitinib plus gemcitabine treatment, 142 (100 up- and 42 down-regulated genes, listed in Supporting Information; Tables S3A and S3B, respectively) were specific to this treatment, while after gemcitabine or masitinib monotherapies, 818 and 201 genes were deregulated, respectively (Figure 4B). When considering these specific combination-regulated genes, no pathway was found to be significantly over-represented among the up-regulated genes (see Supporting Information; Table S2). Among the down-regulated genes, one oncogenic pathway emerged as the most significantly over represented, the Wnt/β-catenin signalling (*p*<0.001). Three other pathways which were altered to a lesser extent included: ERK/MAPK signalling, CDK5 signalling, and PI3K/AKT signalling (*p* = 0.016, 0.025, 0.039, respectively).

## Discussion

The pancreatic tumour cell lines used in this study were selected for their different sensitivities to standard gemcitabine chemotherapy. BxPC-3 and Capan-2 cell growth was efficiently inhibited by gemcitabine, while Mia Paca-2 and Panc-1 cells were resistant. None of the cell lines, including those expressing c-Kit and PDGFRα or β, showed sensitivity to masitinib monotherapy. Of the tyrosine kinases strongly expressed in all four cell lines, masitinib inhibits Lyn (IC_50_ of 400 nM), and to a lesser extent FGFR3 (IC_50_ 2.5 µM) [14]. This suggests that proliferation of these cell lines does not depend significantly upon the major kinase targets of masitinib. The mechanisms leading to gemcitabine resistance in pancreatic cancer are often associated with FAK and SFK. However, in accordance with masitinib's pharmacological profile [14], the observed resensitisation activity of masitinib is not due to direct inhibition of these targets, but more likely results from a complex interplay of factors. Indeed, preliminary data show that despite masitinib being inactive against purified FAK [14], 1 µM of masitinib is capable of reducing FAK phosphorylation in a cell-based assay (our unpublished results). Another possible mechanism of chemoresistance is impaired drug delivery [23]. Olive et al. have demonstrated that the Hedgehog (Hh) signalling pathway has a role in the delivery of chemotherapeutic agents in a mouse model of pancreatic ductal carcinoma [24]. Therefore, additional as yet uncharacterised targets of masitinib may be involved in the molecular mechanism underlying its synergy with gemcitabine. Using a kinome screening approach, J. Iovanna's laboratory has identified kinases involved in the resistance of pancreatic cancer cells to gemcitabine [18]. Among them MAPKAP1/RSK2/ISPK, MAK, PAK4, ADRBK1/GRK2 and PIK3CG were the most active, while SRC inhibition did not enhance the response of cells to gemcitabine, similar to our results with dasatinib. Future work will test the activity of masitinib on these kinases.

Analysis of the transcriptome of gemcitabine-resistant Mia Paca-2 cells revealed differences in up- and down-regulated genes unique to the masitinib plus gemcitabine combination. The most significantly altered pathway involved genes associated with Wnt/β-catenin signalling, a pathway that regulates cell proliferation, differentiation and stem cell renewal [25]. This pathway is involved in pancreatic development and re-activation of this signalling system has been implicated in pancreatic carcinoma with reported nuclear localisation of the downstream effector β-catenin [25]. Down-regulation of genes involved in this signalling pathway by a combination of masitinib plus gemcitabine, may therefore contribute to accelerated death in Mia Paca­2 cells as compared to gemcitabine monotherapy. Hence, it will be important to determine changes in activation, stabilisation and subcellular localisation of β-catenin in Mia Paca-2 cells following treatment with the drug combination. Other down-regulated kinase-associated pathways warranting further investigation included ERK/MAPK signalling, CDK5 signalling and PI3K/AKT signalling.

The efficacy of TKI therapy has been previously evaluated in an orthotopic nude mouse model of human pancreatic cancer, both as monotherapy and as combination therapy with gemcitabine. The inhibitors investigated were the BCR-ABL/c-Kit/PDGFRβ inhibitor imatinib (Glivec, STI571) [26], the EGFR/VEGFR/PDGFR inhibitor AEE-788 [27], and the SFK/ABL inhibitor dasatinib (BMS-354825) [28]. Those preclinical studies demonstrated increased efficiency of gemcitabine when used in combination with kinase inhibitors, resulting mainly in extended survival and inhibition of metastasis. This supports the general interest of using TKIs in combination therapy with gemcitabine. However, under the conditions of this *in vitro* study we were unable to re-sensitise resistant Mia Paca-2 cells to gemcitabine when used in combination with dasatinib or imatinib, in contrast to our findings for masitinib (Figure 2D). One interpretation of these results is that the combination of masitinib plus gemcitabine might be more potent in human pancreatic cancer than other TKIs, particularly in cases of cancers that relapse after a first line of treatment. Additionally, many of these inhibitors, including dasatinib and imatinib, have been associated with cardiotoxicity [29]. Conversely, the accumulated clinical experience of masitinib (>485 patients exposed) has revealed no evidence of cardiotoxicity in humans; consistent with its known low cardiac risk pharmacological profile [14].

In summary, combined treatment with masitinib plus gemcitabine resulted in resensitisation of resistant pancreatic cell lines *in vitro*. This chemosensitisation may allow lower concentrations of gemcitabine to be used, thereby reducing the risk of toxicity or increasing the available efficacy at standard gemcitabine doses. Such synergy was not observed with BxPC-3 and Capan-2 cells, possibly because of the already strong cytotoxicity of gemcitabine on these cell lines. In this study, masitinib was used at 5 and 10 µM over a 72-hour incubation time. These conditions do not necessarily reflect those to be used in the clinical setting, but rather demonstrate the concept. Pharmacokinetic data from previous clinical studies show that at typical masitinib doses (≤12 mg/kg/day), concentrations of 2 µM are achievable *in vivo*. However, repetition of the proliferation assays at 1 and 2 µM failed to reproduce the observed resensitisation (data not shown). For this reason, the *in vivo* antiproliferative activity of masitinib was explored in a Nog­SCID mouse model of human pancreatic cancer. As expected, gemcitabine monotherapy efficiently reduced tumour growth compared to the control, while masitinib monotherapy only weakly inhibited tumour growth. The combination of masitinib plus gemcitabine also reduced tumour growth and showed a possible (statistically non-significant) improvement in tumour inhibition as compared to gemcitabine monotherapy. These results tentatively confirm the hypothesis that masitinib can enhance the antiproliferative activity of gemcitabine *in vivo* and provide supporting evidence for the *in vitro* assay results. However, further confirmation that these proof-of-concept results are of clinical relevance is evidenced by a recent phase 2 study (the results of which postdate the data reported here), in which patients with advanced pancreatic cancer who received a combination of masitinib (9 mg/kg/day) plus gemcitabine showed significantly improved median time-to-progression compared to patients treated with gemcitabine alone [30].

The preclinical data reported here establish the proof-of-concept that masitinib can reverse resistance to chemotherapy in pancreatic tumour cell lines. Masitinib used in combination with gemcitabine has promising potential in the treatment of pancreatic cancer, particularly in cases where the tumour has become refractory to conventional chemotherapy.

## Supporting Information

Table S1 Primer sequences used for kinase gene expression profile.
(0.07 MB DOC)

Table S2 Pathways significantly over represented in the list of genes differentially expressed between the treated and untreated cell lines. Excel file format.
(0.04 MB XLS)

Table S3 Genes differentially and significantly up-regulated/down-regulated in Mia Paca-2 cells treated with masitinib plus gemcitabine.
(0.19 MB DOC)
