# Supplementary material for: Masitinib Combined with Standard Gemcitabine Chemotherapy: In Vitro and In Vivo Studies in Human Pancreatic Tumour Cell Lines and Ectopic Mouse Model
Source: PLoS One. 2010 Mar 4;5(3):e9430. doi: 10.1371/journal.pone.0009430 (PMC2832006; doi:10.1371/journal.pone.0009430)
Supplement: Table S1 — Primer sequences used for kinase gene expression profile. (0.07 MB DOC) [file pone.0009430.s001.doc]

**Table S1. Primer sequences used for kinase gene expression profile.**

| **Accession number** | **Gene** | **Sequence 5’-3’** | **Localisation** | **PCR product**  **size (bp)** | **Annealing**  **temperature** |
| --- | --- | --- | --- | --- | --- |
|  |  |  |  |  |  |
| NM 000222.2  NM 001093772.1 | KIT | TGCTGATTGGTTTCGTAATCG | 1574-1594 | 456 | 54°C |
|  |  | CACCATAGCAACAATATTCTG | 2029-2009 |  |  |
| NM 006206.3 | PDGFRα | TTCTGAACTCACGGTGGCTGCT | 1566-1587 | 423 | 54°C |
|  |  | GCTCCCAGCAAGTTTACAATGT | 1988-1967 |  |  |
| NM 006209.3 | PDGFRβ | TCACCATTCCATGCCGAGTAACAG | 433-457 | 391 | 54°C |
|  |  | ATGGAGCGGATGTGGTAAGGCATA | 824-801 |  |  |
| NM 201284.1  NM 005228.3  NM 201282.1 | EGFR  (erbB1) | ACAGCTATGAGATGGAGGAAGACG | 941-964 | 560 | 56°C |
|  |  | CTTGCAGCTGTTTTCACCTCTGTT | 1500-1477 |  |  |
| NM 001005862.1  NM004448.2 | erbB2 | TCCTACATGCCCATCTGGAAGT | 1825-1846 | 606 | 59°C |
|  |  | GACATGGTCTAAGAGGCAGCCATA | 2430-2407 |  |  |
| NM 002253.1 | VEGFR | GCGGCAAATGTGTCAGCTTTGT | 1561-1582 | 413 | 59°C |
|  |  | TGCCTGACCACGCAATGTCTTT | 1973-1952 |  |  |
| NM 198291.1  NM 005417.3 | SRC | GGTGCAGTTGTATGCTGTGGTTTC | 975-998 | 321 | 59°C |
|  |  | GTCCACTTGATGGGGAATTTGGCA | 1294-1272 |  |  |
| NM 002350.1 | LYN | AGGGAGAACTAATGCCGACGTGAT | 1335-1357 | 481 | 59°C |
|  |  | CTTCTCAAACGGCTGCACTCAGAT | 1816-1793 |  |  |
| NM 153831.2  NM 005607.3 | FAK | CAGTCCGAGGTCCAGCGAAG | 2163-2182 | 325 | 56°C |
|  |  | CATCAGATGGGTTGGCAACAC | 2404-2424 |  |  |
| NM 022965.1  NM 000142.2 | FGFR3 | CAACTGCACACACGACCTGTACAT | 2151-2174 | 386 | 59°C |
|  |  | AGCTCTGTGTAGCTGTCTCTCCAT | 2537-2513 |  |  |
| NM 000181.2 | GUS | CGCCCTGCCTATCTGTATTC | 962-981 | 91 | 59°C |
|  |  | TCCCCACAGGGAGTGTGTAG | 1033-1052 |  |  |
