# Supplementary material for: Masitinib Combined with Standard Gemcitabine Chemotherapy: In Vitro and In Vivo Studies in Human Pancreatic Tumour Cell Lines and Ectopic Mouse Model
Source: PLoS One. 2010 Mar 4;5(3):e9430. doi: 10.1371/journal.pone.0009430 (PMC2832006; doi:10.1371/journal.pone.0009430)
Supplement: Table S3 — Genes differentially and significantly up-regulated/down-regulated in Mia Paca-2 cells treated with masitinib plus gemcitabine. (0.19 MB DOC) [file pone.0009430.s003.doc]

**Table S3A.** Genes differentially and significantly up-regulated in Mia Paca-2 cells treated with masitinib plus gemcitabine.

| Symbol | Gene Name | | GeneBank Accession | Fold Change |
| --- | --- | --- | --- | --- |
| LOC647 | Hypothetical LOC647070 | AK001442 | | 2.03 |
| ZNF652 | Zinc finger protein 652 | NM_014897 | | 2.79 |
| HNMT | Histamine N-methyltransferase | BC005907 | | 2.13 |
| SERP1 | Stress-associated endoplasmic reticulum protein 1 | AI580135 | | 2.43 |
| SNX10 | Sorting nexin 10 | NM_013322 | | 2.04 |
| DLL1 | Delta-like 1 (Drosophila) | AF196571 | | 2.19 |
| ZNF81 | Zinc finger protein 81 | AI434443 | | 2.07 |
| MFAP3L | Microfibrillar-associated protein 3-like | NM_021647 | | 2.07 |
| EHHAD | Enoyl-Coenzyme A, hydratase/3-hydroxyacyl Coenzyme A dehydrogenase | NM_001966 | | 2.1 |
| CMAS | Cytidine monophosphate N-acetylneuraminic acid synthetase | NM_018686 | | 2.07 |
| PDCD6 | Programmed cell death 6 | BC020552 | | 2.1 |
| CHAC2 | ChaC, cation transport regulator homolog 2 (*E, coli*) | AI191897 | | 2.1 |
| HACE1 | HECT domain and ankyrin repeat containing, E3 ubiquitin protein ligase 1 | AB037741 | | 2.03 |
| RAB8B | RAB8B, member RAS oncogene family | AI807023 | | 2.07 |
| TPM1 | Tropomyosin 1 (alpha) | Z24727 | | 2.06 |
| BLZF1 | Basic leucine zipper nuclear factor 1 | NM_003666 | | 2.11 |
| KIAA1715 | KIAA1715 | AI814587 | | 2.31 |
| PRKAA1 | Protein kinase, AMP-activated, alpha 1 catalytic subunit | AI935917 | | 2.13 |
| C18orf56 | Chromosome 18 open reading frame 56 | AW291159 | | 2.33 |
| IFT80 | Intraflagellar transport 80 homolog (*Chlamydomonas*) | AI473255 | | 2.19 |
| LOC147299 | Hypothetical protein LOC147299 | AI588962 | | 2.03 |
| BRP44L | Brain protein 44-like | NM_016098 | | 2 |
| AIG1 | Androgen-induced 1 | AF151861 | | 2.1 |
| SESN1 | Sestrin 1 | NM_014454 | | 2.16 |
| DLX4 | Distal-less homeobox 4 | NM_001934 | | 2.11 |
| C10orf97 | Chromosome 10 open reading frame 97 | NM_024948 | | 2.27 |
| GRAMD1C | GRAM domain containing 1C | NM_017577 | | 2.64 |
| PDK3 | Pyruvate dehydrogenase kinase, isozyme 3 | NM_005391 | | 2.17 |
| EFCAB2 | EF-hand calcium binding domain 2 | BC002836 | | 2.03 |
| C6orf115 | Chromosome 6 open reading frame 115 | AF116682 | | 2.22 |
| ARL6IP6 | ADP-ribosylation-like factor 6 interacting protein 6 | AL581082 | | 2.06 |
| LRRC57 | Leucine rich repeat containing 57 | AW135740 | | 2 |
| GMNN | Geminin, DNA replication inhibitor | NM_015895 | | 2.03 |
| MEF2B | Myocyte enhancer factor 2B | BC004449 | | 2.04 |
| RDM1 | RAD52 motif 1 | AA761980 | | 3.12 |
| PGGT1B | Protein geranylgeranyltransferase type I, beta subunit | BF029960 | | 2.07 |
| PMS1 | PMS1 postmeiotic segregation increased 1  (*S, cerevisiae*) | BC008410 | | 2.45 |
| PPP2R2C | Protein phosphatase 2 (formerly 2A), regulatory subunit B, gamma isoform | AI669212 | | 2.3 |
| CD40 | CD40 molecule, TNF receptor superfamily member 5 | NM_001250 | | 2.07 |
| IDH2 | Isocitrate dehydrogenase 2 (NADP+), mitochondrial | AU151428 | | 2.2 |
| SENP8 | SUMO/sentrin specific peptidase family member 8 | AW173305 | | 2.06 |
| CABYR | Calcium binding tyrosine-(Y)-phosphorylation regulated | NM_012189 | | 2.06 |
| OXR1 | Oxidation resistance 1 | AL541048 | | 2.33 |
| MEA1 | Male-enhanced antigen 1 | NM_014623 | | 2.1 |
| ZUFSP | Zinc finger with UFM1-specific peptidase domain | AI927382 | | 2 |
| C16orf61 | Chromosome 16 open reading frame 61 | NM_020188 | | 2.1 |
| ENOX2 | Ecto-NOX disulfide-thiol exchanger 2 | S72904 | | 2.03 |
| ZNF138 | Zinc finger protein 138 | AA114243 | | 2.08 |
| ELAC1 | ElaC homolog 1 (*E, coli*) | AI669235 | | 2.1 |
| MND1 | Meiotic nuclear divisions 1 homolog (*S, cerevisiae*) | AY028916 | | 2.19 |
| PHF20L1 | PHD finger protein 20-like 1 | AW612407 | | 2.1 |
| RPS27L | Ribosomal protein S27-like | BC003667 | | 2.07 |
| KIF1A | Kinesin family member 1A | AL533416 | | 2.22 |
| C15orf57 | Chromosome 15 open reading frame 57 | AW575737 | | 2.14 |
| BCHE | Butyrylcholinesterase | NM_000055 | | 2.22 |
| C21orf45 | Chromosome 21 open reading frame 45 | AW151538 | | 2.11 |
| DNAJC18 | DnaJ (Hsp40) homolog, subfamily C, member 18 | BG168666 | | 2 |
| BPGM | 2.3-bisphosphoglycerate mutase | NM_001724 | | 2.58 |
| WIPF3 | WAS/WASL interacting protein family, member 3 | AI807950 | | 2.35 |
| C1orf97 | Chromosome 1 open reading frame 97 | BC005997 | | 2.16 |
| CLEC2B | C-type lectin domain family 2, member B | BC005254 | | 2.1 |
| DISP1 | Dispatched homolog 1 (*Drosophila*) | AI677948 | | 2.83 |
| BTN3A2 | Butyrophilin, subfamily 3, member A2 | BC002832 | | 2.3 |
| HFE | Hemochromatosis | BG402460 | | 2.93 |
| ZNF394 | Zinc finger protein 394 | AK022360 | | 2.28 |
| RHEBL1 | Ras homolog enriched in brain like 1 | BC014155 | | 2.31 |
| KRCC1 | Lysine-rich coiled-coil 1 | AK025986 | | 2.01 |
| AFAP1L1 | Actin filament associated protein 1-like 1 | H28999 | | 2.27 |
| SFN | Stratifin | X57348 | | 2.13 |
| DNAJC10 | DnaJ (Hsp40) homolog, subfamily C, member 10 | BG168666 | | 2.07 |
| GNL3L | Guanine nucleotide binding protein-like 3 (nucleolar)-like | NM_019067 | | 2.77 |
| C11orf74 | Chromosome 11 open reading frame 74 | AA535128 | | 2.1 |
| OIP5 | Opa interacting protein 5 | BE045993 | | 2.23 |
| SNAPC3 | Small nuclear RNA activating complex, polypeptide 3, 50kDa | U71300 | | 2.17 |
| ATG10 | ATG10 autophagy related 10 homolog (*S, cerevisiae*) | AL136912 | | 2.41 |
| SRFBP1 | Serum response factor binding protein 1 | AI391443 | | 2.2 |
| ZNF44 | Zinc finger protein 44 | AI758888 | | 2.11 |
| MOBKL3 | MOB1, Mps One Binder kinase activator-like 3 (yeast) | NM_015387 | | 2.06 |
| FAM73A | Family with sequence similarity 73, member A | BE645144 | | 2.19 |
| CHMP4B | Chromatin modifying protein 4B | AW299290 | | 2.14 |
| ATP6V1C1 | ATPase, H+ transporting, lysosomal 42kDa, V1 subunit C1 | AW024925 | | 2.01 |
| RAB7L1 | RAB7, member RAS oncogene family-like 1 | BC002585 | | 2.06 |
| LYRM2 | LYR motif containing 2 | AV682940 | | 2.22 |
| TMEM17 | Transmembrane protein 17 | AA084725 | | 2.04 |
| MAD2L1 | MAD2 mitotic arrest deficient-like 1 (*yeast*) | NM_002358 | | 2.04 |
| ZFAND2A | Zinc finger, AN1-type domain 2A | AI984061 | | 2.03 |
| C9orf80 | Chromosome 9 open reading frame 80 | AF161411 | | 2.23 |
| NUDT15 | Nudix (nucleoside diphosphate linked moiety X)-type motif 15 | NM_018283 | | 2.06 |
| PPIL5 | Peptidylprolyl isomerase (cyclophilin)-like 5 | AA742244 | | 2.01 |
| WBP4 | WW domain binding protein 4 (formin binding protein 21) | AI734228 | | 2.04 |
| METTL4 | Methyltransferase like 4 | AA764787 | | 2.08 |
| TTF1 | Transcription termination factor, RNA polymerase I | NM_007344 | | 2.16 |
| LOC285550 | Hypothetical protein LOC285550 | AW439843 | | 2.33 |
| MRTO4 | mRNA turnover 4 homolog (S, cerevisiae) | BG107419 | | 2.04 |
| ADSSL1 | Adenylosuccinate synthase like 1 | BF593252 | | 2.38 |
| ADK | Adenosine kinase | NM_001123 | | 2.04 |
| AS3MT | Arsenic (+3 oxidation state) methyltransferase | AF226730 | | 2.01 |
| RP2 | Retinitis pigmentosa 2 (X-linked recessive) | NM_006915 | | 2.1 |
| GLIPR2 | GLI pathogenesis-related 2 | H92988 | | 2.13 |
| PSMB9 | Proteasome (prosome, macropain) subunit, beta type, 9 (large multifunctional peptidase 2) | NM_002800 | | 2.06 |

100 up

**Table S3B:** Genes differentially and significantly down-regulated in Mia Paca-2 cells treated with masitinib plus gemcitabine.

| Symbol | Gene Name | GeneBank Accession | Fold Change |
| --- | --- | --- | --- |
| VIM | Vimentin | AI520969 | -2.46 |
| HEXIM1 | Hexamethylene bis-acetamide inducible 1 | AW193511 | -2.06 |
| ANXA1 | Annexin A1 | AU155094 | -5.54 |
| PHLDA1 | Pleckstrin homology-like domain, family A, member 1 | NM_007350 | -2.04 |
| TAF1D | TATA box binding protein (TBP)-associated factor, RNA polymerase I, D, 41 kDa | NM_024116 | -2.16 |
| SNORD79 | Small nucleolar RNA, C/D box 79 | AI056992 | -2.38 |
| OSR1 | Odd-skipped related 1 (Drosophila) | AI569974 | -2.2 |
| KRT80 | Keratin 80 | AL162069 | -2.08 |
| CIRBP | Cold inducible RNA binding protein | AL565767 | -2.35 |
| GAS5 | Growth arrest-specific 5 (non-protein coding) | AW105301 | -2.35 |
| CYP27C1 | Cytochrome P450, family 27, subfamily C, polypeptide 1 | BC039307 | -2.1 |
| CBX6 | Chromobox homolog 6 | NM_014292 | -2.06 |
| UBXN6 | UBX domain protein 6 | AF272894 | -2.04 |
| SYTL1 | Synaptotagmin-like 1 | AI341537 | -2.11 |
| PPP2R4 | Protein phosphatase 2A activator, regulatory subunit 4 | X86428 | -2.06 |
| ITGA3 | Integrin, alpha 3 (antigen CD49C, alpha 3 subunit of VLA-3 receptor) | NM_002204 | -2.01 |
| MYADM | Myeloid-associated differentiation marker | AA909044 | -2.07 |
| RUNX1 | Runt-related transcription factor 1 | D43968 | -2.68 |
| NEBL | Nebulette | NM_006393 | -2.75 |
| DENND5B | DENN/MADD domain containing 5B | AL137364 | -2.04 |
| SLC39A10 | Solute carrier family 39 (zinc transporter), member 10 | AB033091 | -2.41 |
| KCTD15 | Potassium channel tetramerisation domain containing 15 | AI808448 | -2.71 |
| HNRPDL | Heterogeneous nuclear ribonucleoprotein D-like | AB066484 | -2.11 |
| CD97 | CD97 molecule | NM_001784 | -2.13 |
| JAG2 | Jagged 2 | Y14330 | -2.04 |
| TLE3 | Transducin-like enhancer of split 3 (E(sp1) homolog, (*Drosophila*) | BE967118 | -2.69 |
| CITED2 | Cbp/p300-interacting transactivator, with Glu/Asp-rich carboxy-terminal domain, 2 | NM_006079 | -2.62 |
| MKNK2 | MAP kinase interacting serine/threonine kinase 2 | AA404592 | -2.07 |
| SOX12 | SRY (sex determining region Y)-box 12 | NM_006943 | -2.04 |
| FANCF | Fanconi anemia, complementation group F | NM_022725 | -2.13 |
| IRF2BP2 | Interferon regulatory factor 2 binding protein 2 | BF968057 | -2.23 |
| CSNK1E | Casein kinase 1, epsilon | T51255 | -2.01 |
| KAT5 | K(lysine) acetyltransferase 5 | AA886971 | -2.08 |
| SOCS6 | Suppressor of cytokine signaling 6 | NM_004232 | -2.17 |
| MZF1 | Myeloid zinc finger 1 | AF055078 | -2.04 |
| SOX9 | SRY (sex determining region Y)-box 9 | NM_000346 | -2.2 |
| C15orf52 | Chromosome 15 open reading frame 52 | BE673226 | -2.39 |
| DHRS2 | Dehydrogenase/reductase (SDR family) member 2 | AK000345 | -2.16 |
| NFIX | Nuclear factor I/X (CCAAT-binding transcription factor) | AI355848 | -2.03 |
| CXCL2 | Chemokine (C-X-C motif) ligand 2 | M57731 | -2.28 |
| NR2F2 | Nuclear receptor subfamily 2, group F, member 2 | AL554245 | -2.33 |
| EPHB4 | Ephrin receptor B4 | NM_006377 | -2.01 |

Gray background indicates involvement in the Wnt/β-catenin pathway

42 down
